# Survey data on growth companies’ and control group's perceptions on innovative behavior

**DOI:** 10.1016/j.dib.2022.108324

**Published:** 2022-05-29

**Authors:** Arho Suominen, Matti Pihlajamaa

**Affiliations:** 1 VTT Technical Research Centre of Finland, Espoo, Finland; 2 Tampere University, Industrial Engineering and Management, Tampere, Finland

**Keywords:** firm growth, growth expectations, organizational innovation, product innovation, innovation outcomes, market dynamics

## Abstract

The data set contains data collected using a paper format and an online survey. The data includes a sample of 120 Finnish companies. The survey, structured into six sections focuses on the firms’ growth outlooks and the underlying management practices and principles. The data reports the perceptions of top managers on growth, innovativeness, and the ability for renewal. The majority of the data comprises Likert scale questions on respondents’ agreement or disagreement on innovation behaviors. This is complemented with micro-level data based indicator if a company is a growth company, the companies’ growth expectations as percentage, and if COVID-19 has had business impacts. The data description is associated with a codebook and survey form. The data can be used to better understand growth and non-growth companies behaviours and outcomes. The associated material offers an opportunity to replicate the study in different regions.

## Specifications Table

**Table:** 

Subject	Business, Management and decision sciences
Specific subject area	Management of Technology and Innovation
Type of data	Table, Figures
How the data were acquired	*The data were obtained using a questionnaire sent via mail to participants. Participants had the opportunity to return the survey in paper format or fill in the survey via an online survey platform (Webropol). The questionnaire used is attached as an appendix to the paper.*
Data format	*Raw, Analyzed (descriptive statistics)*
Description of data collection	*The data was collected includes information on 1) company being a growth company or not, 2) growth outlook, 3) innovative behavior, 4) managerial behavior, 5) innovation outcomes, and 6) market dynamics.*
Data source location	*Finland*
Data accessibility	Repository name: Zenodo Data identification number: 10.5281/zenodo.5820394 Direct URL to data: https://doi.org/10.5281/zenodo.5820394

## Value of the Data

• Researchers in the field of entrepreneurship are interested in what sets growth companies apart from non-growth companies. Most past research investigates past growth, e.g, [1], [2]. However, there is also interest in understanding differences in firms’ growth expectations, aspirations, and motivation [3], [4], [5]. This dataset covers both aspects and enables multifaceted analyses of firm growth.
• These data may be used to analyse the effects of past growth outcomes on managers’ growth orientation and innovative behaviour. This is valuable for entrepreneurship researchers interested in the temporal dynamics of growth, i.e., how past successes are related to growth-related managerial goals and decisions at a later time.
• These data may be used by researchers in the field of innovation management and strategy to provide insights into the special characteristics of growth companies. The dataset can be used to identify how companies with different growth profiles approach innovation.
• The dataset can be used to explore associations between the antecedents of innovation (organizational behaviours, interactions of firm management, growth-orientation) and innovation outcomes.
• The dataset is valuable for researchers who want to conduct comparative studies related to the differences between growth and non-growth companies and the association between growth-related factors or antecedents of innovation and innovation outcomes in other countries or industries.

## 1 Data Description

This data is associated with an SPSS data file. The file contains responses from 120 valid responses received. The data file contains responses to six sets of questions for the respondents. The data excludes the open-ended responses seen in the questionnaire form as question 7 and the unique id required from online responses as question 1, labelled Growth_Firm and seen in Table 1. The data also contains a dichotomous variable if the respondent was classified as a growth company or a non-growth company using the definition by Eurostat-OECD [6] with both turnover and employee growth as criteria.

**Table 1:** Frequency distributions as of Growth and Non-growth companies in the sample (N=120).

	Frequency	Percent	Valid Percent	Cumulative Percent
Non-growth company	58	48,3	48,3	48,3
Growth company	62	51,7	51,7	100,0
Total	120	100,0	100,0	

**Fig. 1:**
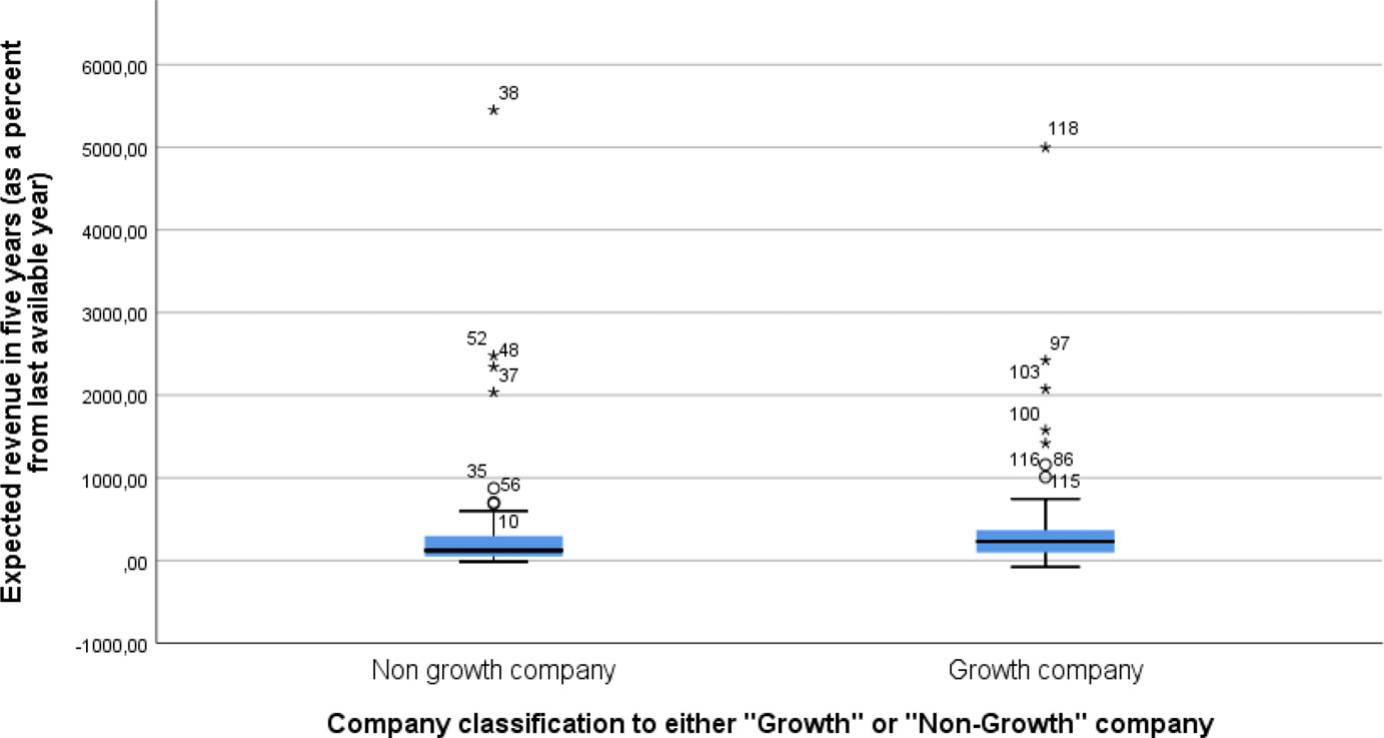
Box plot of expected revenue growth in five years as percentage of last available year. One Growth Company outlier observation (number 91 value 16103,05) excluded from the graph

**Fig. 2:**
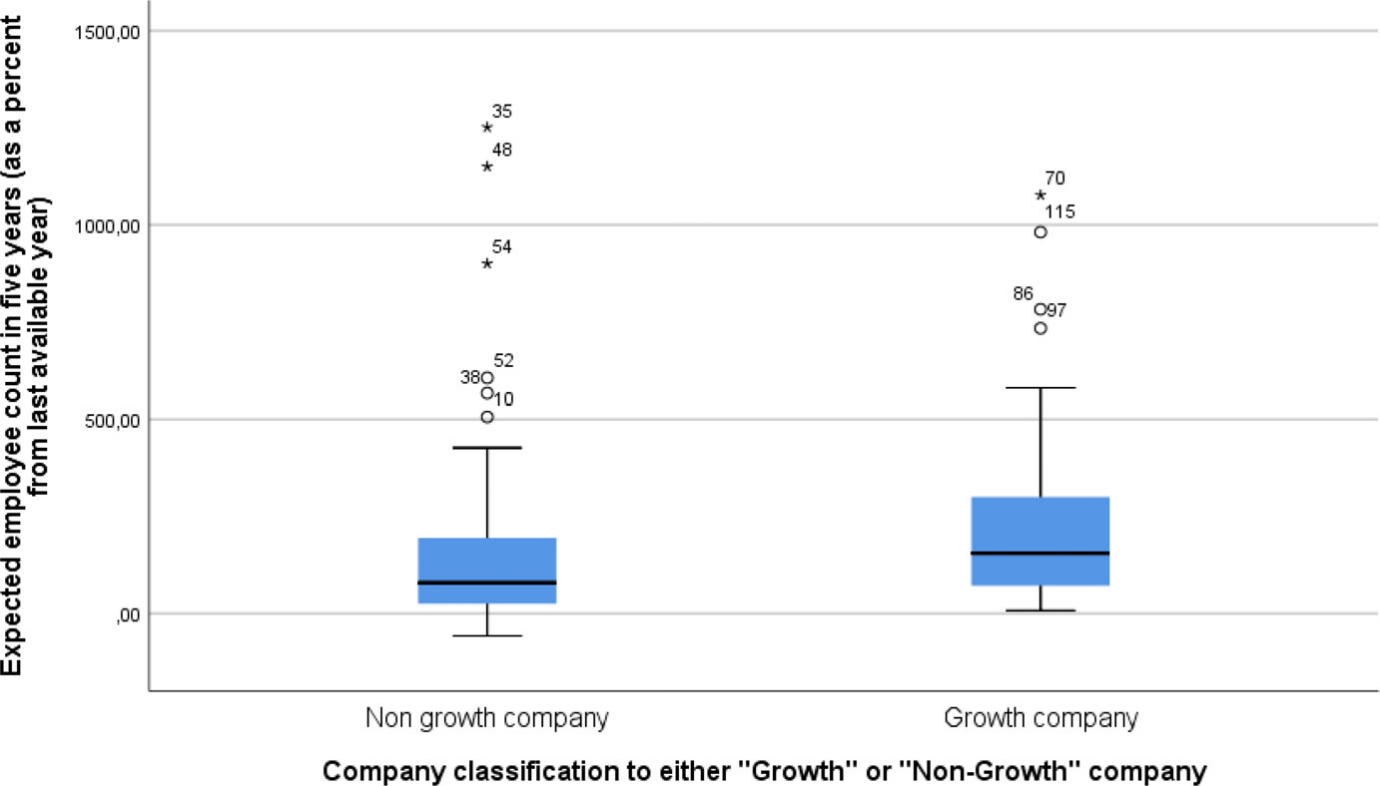
Box plot of expected employee growth in five years as percentage of last available year.

**Fig. 3:**
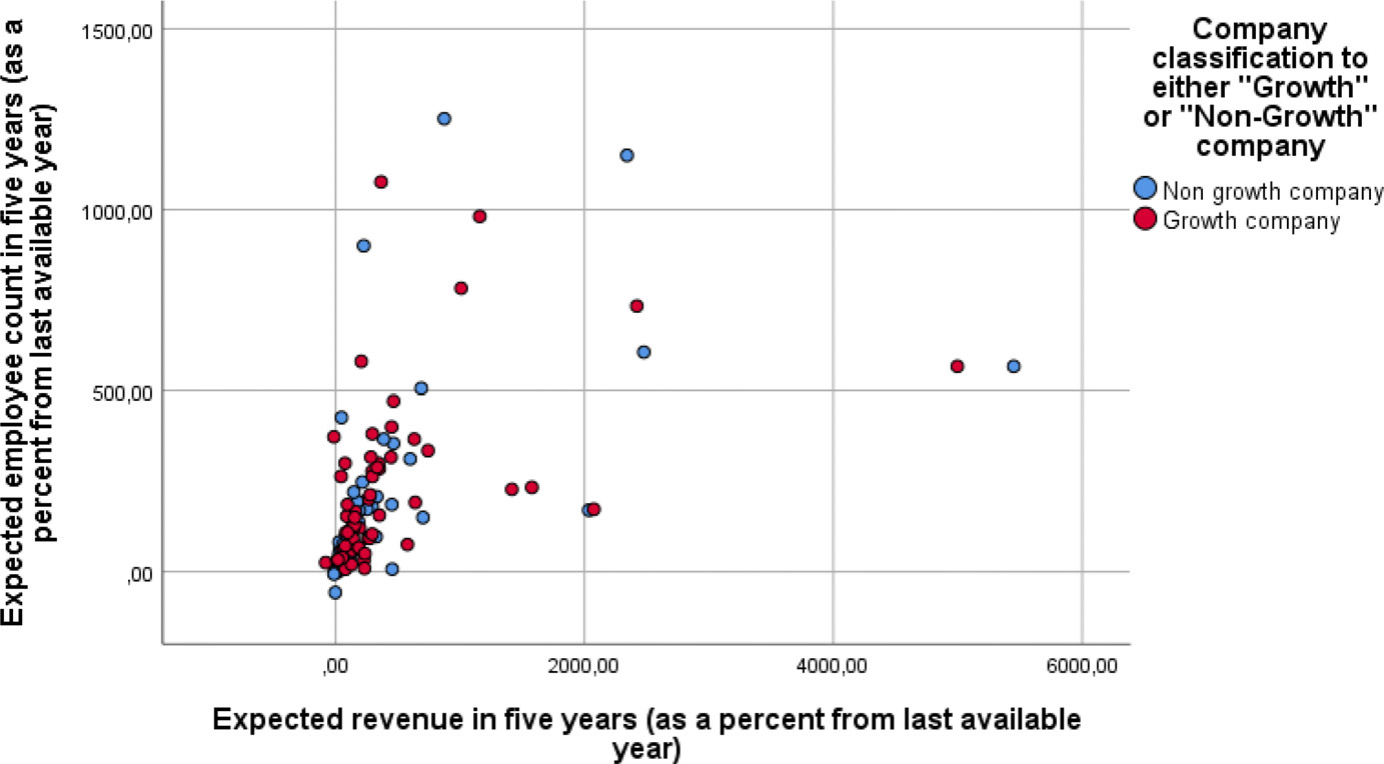
Company growth expectation as percentage overlayed with growth firm status. One Growth Company outlier observation (number 91 value 16103,05) excluded from the graph.

The first set of content questions, adopted from Delmar and Wiklund [7], focuses on companies’ expectations for future growth. The respondents were asked, “[i]f the firm develops the way you would like it to, how much revenue would the firm receive, and how many employees would it have five years ahead? Disregard possible inflation”. The respondents were asked to evaluate “The number of employees in five years” and “Revenue (million €) in five years”. Seen in Fig. 1 and 2, we can see the Box Plot of future expectations for both revenue and employee growth. As seen in both figures, there are a number of outliers where several companies have a highly positive view of future expectations. For these variables, we report the percentage change to revenue and number of employees from the last available year, coded as question_2_row_1_transformed for employee growth and question_2_row_2_transformed for revenue growth and seen in Fig. 3. To test if there are statistically significant differences between the two samples Mann-Whitney U test was run. A Mann-Whitney U test shows that Growth companies (Mdn= 62) have higher than non-growth companies (Mdn= 58) revenue growth (U= 1330.0, p=0.014) and employee growth (U= 1299.0, p=0.009) expectations.

Question three comprised 16 questions focusing on organizational behaviours. The questions originate from Ruvio et al. [8], and they were measured with a 5-point Likert scale from Strongly disagree to Strongly agree. The original question list by Ruvio et al. [8] comprises 21 questions that address five dimensions of innovativeness: creativity (crt), openness (opn), future orientation (ftr), risk-taking (rsk), and proactiveness (prc). Five questions were left out of our questionnaire. The remaining set of questions included questions that were thematically similar to those omitted. Each question has 120 observations. The percentage frequency distribution across the Likert-scale and the associated dimensions of innovativeness can be seen in Table 2. The questions are coded in the data as question_3_row_1 to question_3_row_16.

**Table 2:** Frequency distributions as percentage (N=120) for question 3 rows one to sixteen.

	Strongly disagree	Disagree	Neither agree or disagree	Agree	Strongly agree
Employees are encouraged to be creative (crt)	0,0%	5,0%	15,0%	55,8%	24,2%
Managers are expected to be creative problem solvers (crt)	0,0%	0,8%	8,3%	44,2%	46,7%
Employees' ability to function creatively is respected (crt)	0,0%	1,7%	7,5%	49,2%	41,7%
We are constantly looking for ways to develop and offer new or improved products and services (crt)	0,8%	1,7%	5,8%	52,5%	39,2%
Assistance in developing new ideas is readily available (opn)	0,0%	4,2%	18,3%	60,0%	17,5%
Our organization is open and responsive to changes (opn)	0,0%	3,3%	15,0%	52,5%	29,2%
Managers here are always searching for fresh, new ways of looking at problems (opn)	0,0%	1,7%	14,2%	56,7%	27,5%
Our organization has a clear and inspiring set of future goals (ftr)	0,0%	3,3%	15,8%	45,0%	35,8%
We have ensured that all managers and employees share the same vision of the future (ftr)	0,0%	1,7%	20,0%	56,7%	21,7%
All departments and employees share a clear vision of the future (ftr)	0,8%	9,2%	34,2%	43,3%	12,5%
We believe that higher risks are worth taking for high payoff (rsk)	3,3%	15,8%	32,5%	30,0%	18,3%
We encourage innovative initiatives, knowing well that some will fail (rsk)	0,0%	5,8%	25,8%	48,3%	20,0%
We do not like to “play it safe” (rsk)	3,3%	20,8%	34,2%	30,0%	11,7%
Managers are constantly seeking new opportunities for the organization (prc)	1,7%	10,8%	20,0%	44,2%	23,3%
Managers take the initiative in an effort to shape the environment to the organization's advantage (prc)	0,8%	2,5%	15,0%	53,3%	28,3%
Managers usually take the initiative by introducing new administrative techniques (prc)	0,8%	9,2%	33,3%	40,8%	15,8%

**Fig. 4:**
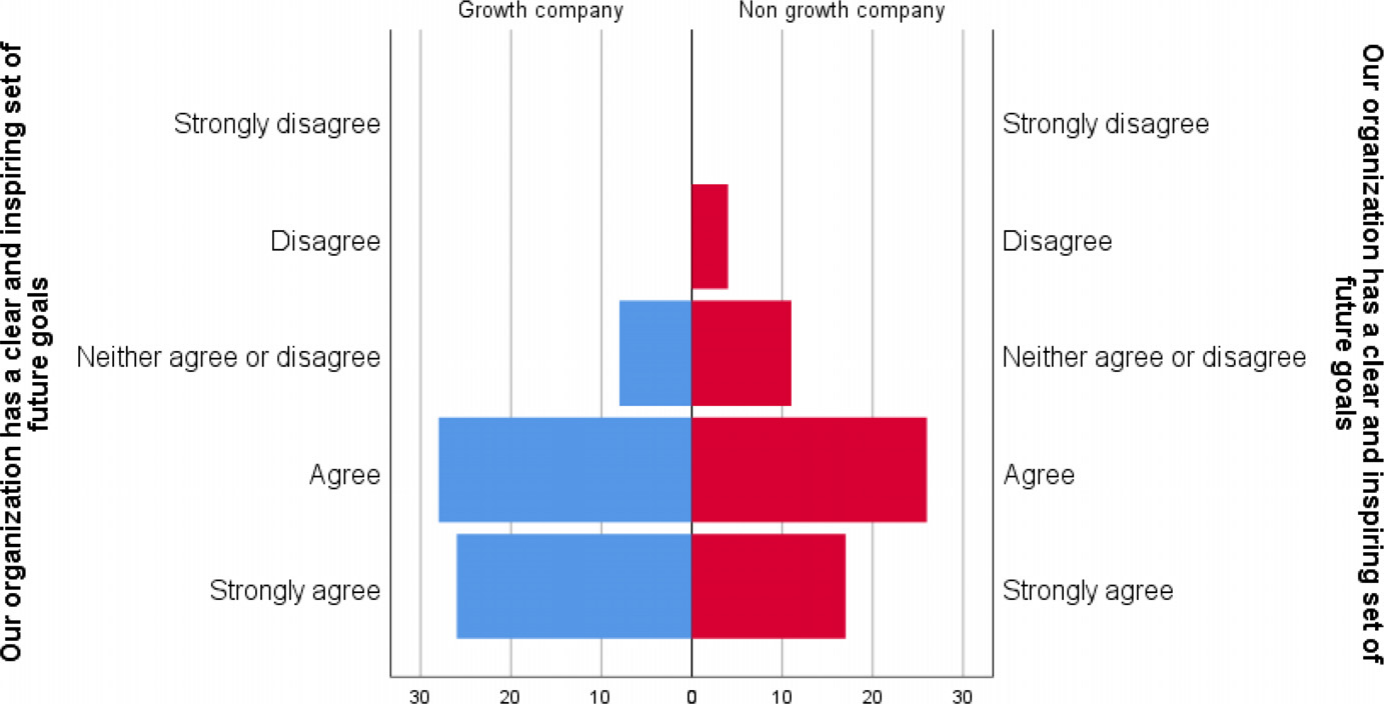
Pyramid historgram of responses to question “Our organization has a clear and inspiring set of future goals” clustered with company category being Growth or Non-growth company (N=120).

The Likert scale responses are skewed towards an agreement with the statement. For many of the statements, the strongly disagree response did not receive any responses. This said, focusing on the core variable of growth of non-growth companies, we can see differences in the responses. Particularly in that the growth companies skew even more towards agreement and non-growth companies show more disagreement. A case in point is the question is “Our organization has a clear and inspiring set of future goals” seen in Fig. 4. This suggests that the data has value in drawing out differences between the two groups.

We investigated which organizational behavior related questions have statistically significant differences between the two samples using the Mann-Whitney U test. A Mann-Whitney U test shows that growth companies (Mdn= 62) are more often than non-growth companies (Mdn= 58) looking for ways to develop and offer new or improved products and services (U= 1384.5, p=0.015). In addition, growth companies (Mdn= 62) are more often than non-growth companies (Mdn= 58) have internal assistance in developing new ideas is readily (U= 1457.2, p=0.042). Growth companies (Mdn= 62) more often than non-growth companies (Mdn= 58) have a clear and inspiring set of future goals (U= 1449.0, p=0.048). Finally, growth companies (Mdn= 62) are more often than non-growth companies (Mdn= 58) are defined by managers usually take the initiative by introducing new administrative techniques (U= 1297.5, p=0.005). For all other questions, we are unable to reject the null hypothesis.

The second set of questions, coded as question_4_row_1 to question_4_row_4, focuses on the interactions of firm management. Two of the questions, “Our company's top management frequently has discussions on renewal, innovation, and growth with managers from other companies” and “Interactions with managers from other companies have helped us build our capabilities and skills”, were adapted from Bruneel et al. [4]. These questions were modified to focus on innovation and growth and emphasize managers’ interactions with their peers. The other two questions are not based on previous studies. The data contains valid responses for all 120 companies for each subquestion in the set. The frequency distribution as percentage is given in Table 3. When focusing on which of the leadership behavior related questions have statistically significant differences between the two samples using the Mann-Whitney U test, we are unable to reject the null hypothesis for any of the four statements made.

**Table 3:** Frequency distributions as percentage (N=120) for question 4 rows one to four.

	Strongly disagree	Disagree	Neither agree or disagree	Agree	Strongly agree
Our company's top management frequently has discussions on renewal, innovation, and growth with managers from other companies	0,8%	15,8%	25,0%	44,2%	14,2%
We have learned important new information on markets, technologies, and administration from interactions with managers from other companies	3,3%	18,3%	27,5%	39,2%	11,7%
Interactions with managers from other companies have helped us build our capabilities and skills	5,0%	20,0%	24,2%	41,7%	9,2%
Active discussions with managers from other companies have generated collaborations leading to new innovations	5,0%	19,2%	25,0%	37,5%	13,3%

The third set of questions in the survey, modified from Jansen et al. [9], focused on innovation outcomes. The questions, labelled question_5_row_1 to question_5_row_10, asked the respondents to evaluate both the type of innovation outcomes and the markets the company is looking towards serving. The original question list by Jansen et al. [9] comprises 14 questions that address two dimensions of innovation outcomes: exploratory innovation (explor) and exploitative innovation (exploit). Six questions were left out of our questionnaire. Two original questions, “We develop our business model to stand out from our competitors” and “We use experiments to identify and evaluate new business opportunities”, were added. The respondents answered the questions in Likert-scale and valid responses were received for all 120 respondents on rows one to eight, and for rows nine and ten, 83 responses were received. The frequency of responses and the associated dimensions of innovation outcomes are seen in Table 4 as percentage shares. We examined which of the innovation outcome-related questions have statistically significant differences between the two samples using the Mann-Whitney U test. The test shows that growth companies (Mdn= 62) are more often than non-growth companies (Mdn= 58) looking for ways to develop business models to stand out from our competitors (U= 1331.0, p=0.008). For all other questions, we are unable to reject the null hypothesis.

**Table 4:** Frequency distributions as percentage (N=120) for question 5 rows one to eight and for questions rows nine and ten (N=83).

	Strongly disagree	Disagree	Neither agree or disagree	Agree	Strongly agree
We commercialize products and services that challenge our previous products/services (explor)	4,2%	11,7%	28,3%	39,2%	16,7%
We develop and commercialize products and services that are completely new (explor)	1,7%	11,7%	20,0%	38,3%	28,3%
We frequently utilize new opportunities in new markets (explor)	0,8%	20,8%	35,0%	30,0%	13,3%
We develop our business model to stand out from our competitors (explor)	0,0%	5,8%	19,2%	47,5%	27,5%
We use experiments to identify and evaluate new business opportunities (explor)	0,8%	15,8%	25,8%	40,8%	16,7%
We frequently make small adjustments to our existing products and services (exploit)	0,0%	6,7%	18,3%	43,3%	31,7%
We improve the efficiency of our products/services (exploit)	0,8%	4,2%	22,5%	49,2%	23,3%
We increase economies of scales in existing markets (exploit)	0,8%	11,7%	30,8%	38,3%	18,3%
We introduce improved versions of existing products and services for our local market (exploit)	1,2%	4,8%	27,7%	43,4%	22,9%
Our organization expands our offering for existing clients (exploit)	0,0%	8,4%	12,0%	44,6%	34,9%

Respondents were also asked about the potential changes in their market environment using questions introduced by Jansen et al. [9]. The original question list by Jansen et al. [9] comprises 5 questions. 3 questions were left out of our questionnaire. The remaining questions, coded question_6_row_1 and question_6_row_2, were asked on a Likert-scale, and valid answers were received for all 120 respondents. The frequency distribution for the answers can be seen in Table 5. We tested if there are statistically significant differences between the samples. The Mann-Whitney U test shows that we cannot reject the null hypothesis.

**Table 5:** Frequency distributions as percentage (N=120) for question 6 rows 1 and 2.

	Strongly disagree	Disagree	Neither agree or disagree	Agree	Strongly agree
The market we operate in is undergoing intense changes	0,8%	11,7%	22,5%	38,3%	26,7%
Our clients regularly ask for new products and services	4,2%	10,0%	34,2%	34,2%	17,5%

The data also contains a question on the impacts of the COVID-19 pandemic, labelled question_7_row_1. The respondents answered if the COVID-19 pandemic has had a significant impact on the firm's actions related to the topics mentioned in the previous questions during the previous year. Valid responses were received for all 120 respondents, and the frequency distribution can be seen in Table 6.

**Table 6:** Frequency distributions as percentage (N=120) for question 7 row 1

Has the COVID-19 pandemic has a significant impact on your firm's actions related to the topics mentioned above during the previous year	Yes	**54**
	No	**66**
	**Total**	**120**

**Fig. 5:**
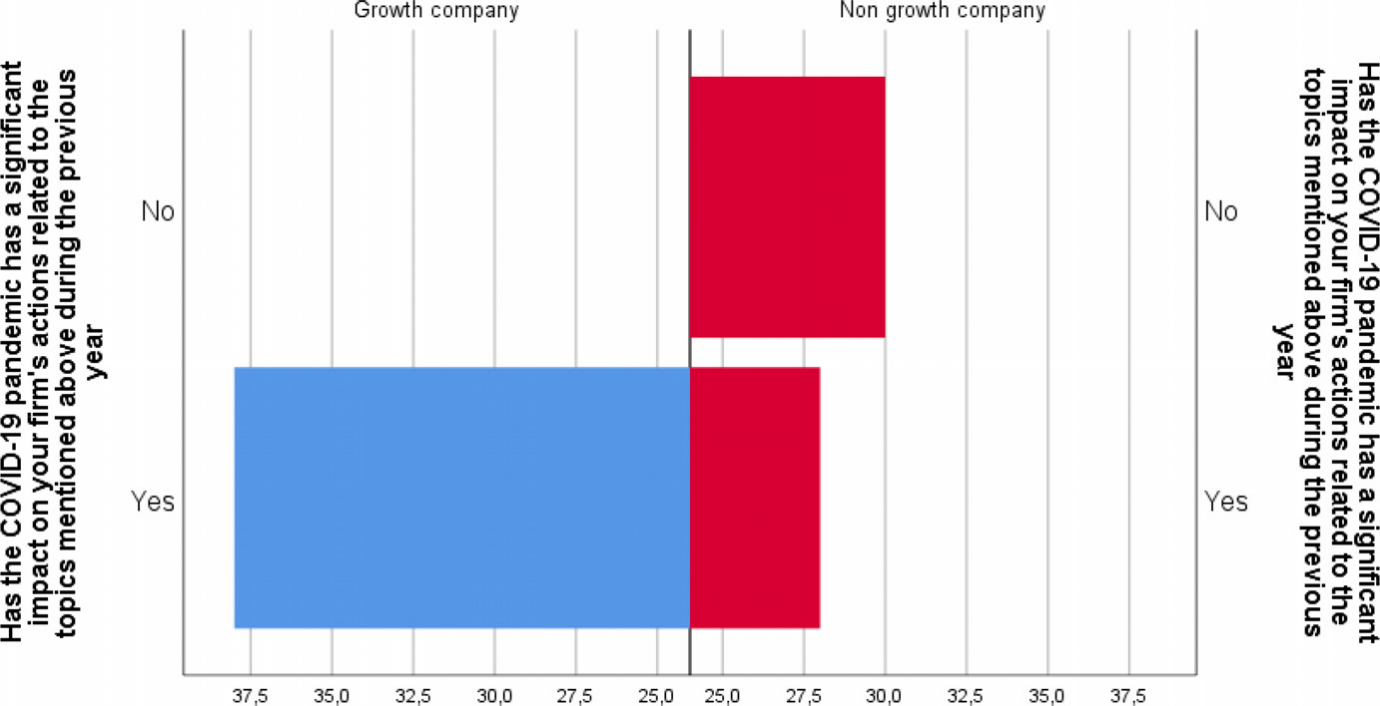
Pyramid histogram of responses to question “Has the COVID-19 pandemic has a significant impact on your firm's actions related to the topics mentioned above during the previous year” clustered with company category being Growth or Non-growth company (N=120).

Also, in this dimension, the data shows important differences between the Growth and Non-Growth companies. Seen in Fig. 5, the number of Growth companies reporting COVID pandemic impacts is higher than that of the non-growth group. After testing if there are statistically significant differences between the growth companies and non-growth companies in experiencing pandemic-related impact, we are, however, unable to reject the null hypothesis.

**Fig. 6:**
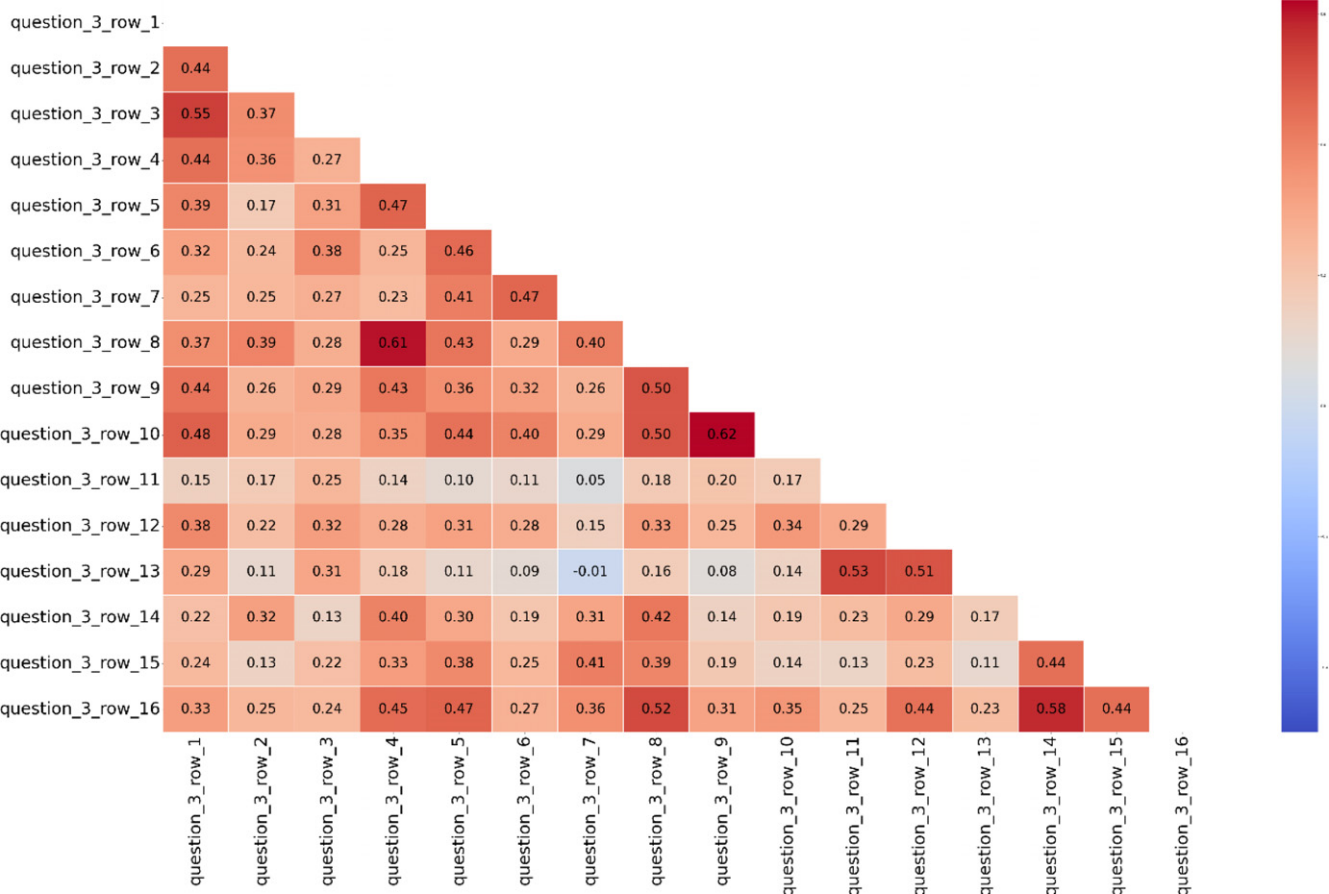
Correlation table for survey questions.

The survey results were analyzed using confirmatory factor analysis. Here, we report the analysis conducted for the questions organizational behaviors, i.e., each innovativeness dimension proposed by Ruvio et al. [8], as it comprises a central part of the data set. Fig. 6 shows the correlation table for each survey question under question three. The questions show correlations between the constructs. To further measure internal consistency, Cronbach Alpha was calculated. For the creativity (crt) construct Cronbach's Alpha was 0.733, openness (opn) 0,71, future orientation (ftr) 0,76, risk-taking (rsk) 0,70 and proactiveness (pcr) 0,74. All of the Cronbach Alpha values are above 0,7 and thus acceptable [10].

**Fig. 7:**
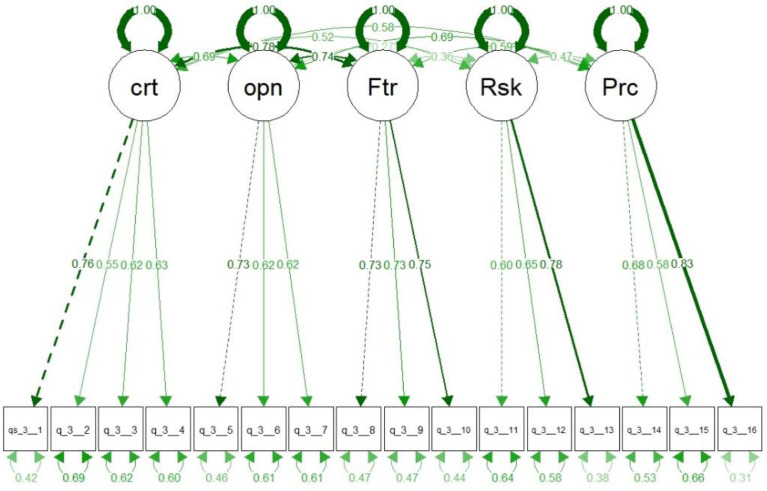
Organizational behaviors CFA model visualization with standardized parameter estimates overlayed.

The CFA was run using R package *lavaan*. Fig. 7 visualizes the CFA model used with the five constructs. The graph is overlayed with the standardized parameter estimates for the model. To estimate the goodness of fit for the model, four fit measures were calculated, namely the comparative fit index (CFI) and the root mean square error of approximation (RMSEA). The CFI value for the model is 0.901 the RMSEA value is 0.074. The fit measures suggest a relatively good fit for the model. CFI is above the commonly used threshold of 0.9, while the RMSEA, above 0.05 but below 0.08, suggesting a mediocre fit. The fit measures suggest that model improvements are possible and further analysis should look into freeing the constraints of the model (e.g. using modification indices analysis) to improve the goodness of fit.

## 2 Experimental Design, Materials and Methods

The sample was created using the Orbis database^1^. The sample was created using four filters. First, companies were selected if they were active or marked as “unknown situation” in the database. Second, the data was limited to only include companies from Finland. Third, the standardised legal form of companies was limited to Public limited company or Private limited company. Finally, we only selected companies with the number of employees being at a minimum of 10 persons. In the Orbis database, this also implies that the number of employees were taken from the last available year and will exclude companies with no recent financial data and public authorities. The filtering process resulted in 17 328 companies being selected from the database. The date of the search was 20.08.2021 at 9:08:55 AM.

Data extracted for the sample included operating revenue, profit/loss before tax, total assets, return on earnings using profit and loss before tax, the number of employees, NACE codes at four-digit level, and contact information for the company. Financial data was downloaded, if available, for the last ten years, the last year being 2020. From the financial variables, we calculated revenue and employee growth for the last three years, namely 2020, 2019 and 2018. For a significant portion of the companies, growth values were unavailable for 2020. This results from the last available data year for a portion of the sample companies is 2019. The NACE four-digit coding was also truncated to two-digit coding. The two-digit coding was also linked with Eurostat [6] industry clustering “High-technology”, “Medium-high technology”, “Knowledge-intensive market services (excluding high-tech and financial services)”, “High-tech knowledge-intensive services”, “Knowledge-intensive financial services” and “Other knowledge-intensive services”. Companies falling outside these Eurostat aggregations were not assigned a coding and excluded for further analysis.

For each company, yearly growth in revenue and employment were calculated from 2017 to 2019. A binary variable for each, revenue growth and employment, was created if the growth was over 20 percent, following the Eurostat-OECD [11] definition of a high-growth enterprise. An indicator of whether the company is a growth firm was created based on both revenue and employment growth of over 20 percent. The data set was also cleaned from companies with missing data. This resulted in a final analysis sample of 2762 companies, 467 growth companies and 2295 non-growth companies.

Propensity score matching was done to match growth companies and non-growth companies. The matching was done using revenue 2019, employees 2019, assets 2019, profit/loss before tax 2019, and NACE as two-digit level. The matching process was calculated using the Python package pymatcher. The average accuracy of the matching is 60,75 percent. A one-to-one match was available for 467 companies paired with one company, making the sample 934. The sample thus contains all growth companies fitting the inclusion criteria and a propensity score-matched sample of non-growth companies.

Using the contact information, companies were sent a survey via mail. The mail was addressed to the Chief Executive Officer of the company. The letter included an invitation letter, survey form and a return envelope. Respondents were offered the option, in the invitation letter, to fill in the survey online using a unique identifier printed in the invitation letter and survey form. Two reminders were sent to respondents via email. In total, 126 responses were received. However, 120 responses were deemed valid, making the response rate 12.8 percent

Survey responses received from the online platform and mail were merged into an SPSS file. From the original Orbis data, we merged the information if the company is either a “growth company” or a “non-growth company” by the definition used.

## Ethics Statements

In gathering and reporting data, the authors have followed the research organizations’ and publishers’ guidelines for ethical behaviour. The paper presents an accurate account of the work carried out, and the data is represented correctly and in its entirety.

Survey respondents were informed of the voluntary and confidential nature of the survey. Respondents were told that all identifying information from the data and the respondents’ identity or company are not included in reporting the research. The respondents were also informed in writing that the findings would be actively communicated in the media, and the data will be used to write scientific articles for international journals. In the case of respondents having questions on the survey, participants were given the researchers' email addresses. Should they have any questions regarding personal data protection or data management, respondents were given an official address at VTT Technical Research Centre of Finland dedicated to responding to responded queries.

## CRediT Author Statement

**Arho Suominen:** Conceptualization, Methodology, Software, Data curation, Writing – original draft preparation, Visualization, Writing – review & editing; **Matti Pihlajamaa:** Conceptualization, Methodology, Data curation, Writing – original draft preparation, Validation, Writing – review & editing.

## Declaration of Competing Interest

The authors declare that they have no known competing financial interests or personal relationships that could have appeared to influence the work reported in this paper.

## Data Availability

What does it take to generate new growth - Survey data on company perceptions on innovative behavior (Original data) (Zenodo).

## Footnotes

1 The data release does not contain any Orbis data, while the sample is drawn from the database. The authors can facilitate linking survey responses to firm-level financial data for researchers with existing Orbis database access.
